# Effect of Shading on Development, Yield and Quality of Bastard Balm Herb (*Melittis melissophyllum* L.)

**DOI:** 10.3390/molecules25092142

**Published:** 2020-05-03

**Authors:** Izabela Szymborska-Sandhu, Jarosław L. Przybył, Ewelina Pióro-Jabrucka, Agata Jędrzejuk, Zenon Węglarz, Katarzyna Bączek

**Affiliations:** 1 Department of Vegetable and Medicinal Plants, Warsaw University of Life Sciences–SGGW, 02-787 Warsaw, Poland; izabela_szymborska@sggw.edu.pl (I.S.-S.); jaroslaw_przybyl@sggw.edu.pl (J.L.P.); ewelina_pioro_jabrucka@sggw.edu.pl (E.P.-J.); zenon_weglarz@sggw.edu.pl (Z.W.); 2 Section of Ornamental Plants, Warsaw University of Life Sciences–SGGW, 02-787 Warsaw, Poland; agata_jedrzejuk@sggw.edu.pl

**Keywords:** light intensity, phenolics, HPLC-DAD, chlorophyll a and b, glandular trichomes, antioxidant enzymes, antioxidant activity

## Abstract

The aim of the study was to assess the effects of *Melittis melissophyllum* shading on its development and accumulation of phenolics. Their content (verbascoside, apiin, luteolin-7-*O*-glucoside, coumarin, 3,4-dihydroxycoumarin, *o*-coumaric acid 2-*O*-glucoside as well as *o*-coumaric, *p*-coumaric, chlorogenic, caffeic, ferulic and cichoric acid) was determined in the herb using HPLC-DAD. The results showed that the content of abovementioned flavonoids and phenolic acids was highest in plants grown under full sunlight. On the other hand, a higher content of coumarin was observed in shaded plants, especially after the seed-setting stage. A similar tendency was noted for the amount of chlorophyll a and b. The content of hydrogen peroxide and malondialdehyde, the activity of polyphenol oxidase and catalase and the antioxidant capacity of plant extracts (measured using DPPH, ABTS and FRAP assays) were found to be the highest in the plants grown in full sunlight. However, the plants grown in moderate (30%) shade were found to thrive best.

## 1. Introduction

Bastard balm (*Melittis melissophyllum* L.) is a perennial plant with wintering rhizomes and hairy stems, with the height of up to 30–50 (80) cm. It has downy, honey-scent foliage and white flowers with a pink lip, clustered at the tips of the stem. The plant grows on moist, rich soil, in partial shade [1,2]. It prefers growing in thermophilic oak woods, while it can also be found in subboreal mixed forests, subcontinental oak–hornbeam forests and thermophilic beech communities. In Poland, *M. melissophyllum* reaches the northern border of its distribution, and grows mainly in the lowlands, in the southern and eastern part of the country [2]. The species is the only representative of the Lamiaceae family legally protected in Poland [3], facing numerous threats. Some of these include changes in the light and hydrological conditions on its habitats related to the natural development of deciduous trees and brushwood along with the age of forests and shadowing of the groundcover [4].

Bastard balm is used as a medicinal and aromatic plant. Its herb, which is collected during the flowering stage, has been long used in traditional European medicine. Due to its antispasmodic and antibacterial properties, the raw material of this plant has been applied to treat digestive problems and skin inflammation and also as a sedative to cure sleeping disorders. In addition, it is used for the treatment of a cold, sore throat and cough [5,6,7]. In the mid-nineteenth century, the leaves of this plant were eaten by the population of central Europe during famine. Currently, *M. melissophyllum* is used for the purpose of aromatizing alcohol beverages and tobacco products [8]. The herb is rich in phenolic compounds, namely coumarin and its derivatives, flavonoids and phenolic acids also contains a small amount of essential oil [9,10,11,12,13,14,15]. The following biologically active compounds have been previously detected in *M. melissophyllum*: flavonoids, including myricetin, quercetin, luteolin, kaempferol, apigenin, as well as some phenolic acids, such as protocatechuic, chlorogenic, *p*-hydroxybenzoic, vanillic, caffeic, syringic, *p*-coumaric, ferulic, sinapic, *o*-coumaric, cinnamic acids [13,14]. Phenolics play an important role in modern high-quality food production due to their antioxidant and antibacterial properties. Nowadays, plant extracts rich in phenolics are employed as natural food and beverages preservatives. They provide multidimensional improvement of stored products by preserving the color, odor or texture as well as by extending their shelf-life [16,17].

The accumulation of phenolics in plants is affected by a number of factors. Among these, the most important are genotype, ontogeny and environmental conditions related to biotic and abiotic factors [18]. Some of these are used to optimize plant growth conditions in order to obtain high-quality raw materials. One of the abiotic factors of high importance is light level. In general, plants can adapt to the changes in light quantity; however, their response is species–specific [19]. In addition, light quality has been reported to affect biosynthesis of phenolic compounds in many plant species. It has been proved that shading decreases the concentration of phenolics and affects their composition in berry skins of *Vitis vinifera* cv. Cabernet Sauvignon [20]. This phenomenon has been also observed to occur in the case of the following species: *Vaccinium myrtillus* L., *Ginkgo biloba* L., *Labisia pumila* Benth. or *Brassica oleracea* var. *sabellica* [21,22,23,24]. Thus, light level may be a suitable tool for modifying the chemical profile of phenolics in plants.

Previous research carried out on *M. melissophyllum* have mainly focused on analyzing the content and composition of biologically active compounds in its herb, with a special focus on its essential oil [9,10,11,12]. However, little is known about the relationship between the accumulation of biologically active compounds in these plants during the ontogenetic development and the environmental factors that affect this process. Since the plants of this species grow naturally in forest habitats, one of the most important factors that are considered to influence its occurrence, development and chemical profile is light access. This should be taken into account especially in the context of active protection of *M. melissophyllum* natural resources, including introduction of the plant into cultivation. Therefore, the aim of our research was to analyze the effects of shading on selected developmental and chemical traits of *M. melissophyllum*, under controlled *ex situ* conditions.

## 2. Results

### 2.1. Phenolics

**Figure 1:**
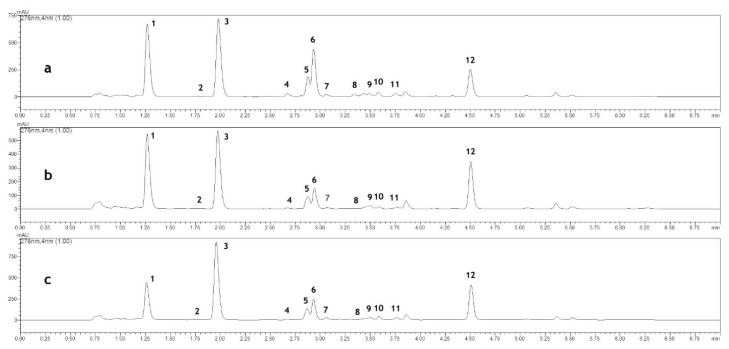
High-performance liquid chromatography (HPLC) chromatogram (monitored at 276 nm) of herb extracts obtained from plants grown: (**a**) in full sunlight; (**b**) in 30% shade; (**c**) in 50% shade. The following compounds were analyzed: (**1**) chlorogenic acid; (**2**) caffeic acid; (**3**) *o*-coumaric acid 2-*O*-glucoside; (**4**) *p*-coumaric acid; (**5**) verbascoside; (**6**) luteolin 7-*O*-glucoside; (**7**) ferulic acid; (**8**) 3,4-dihydroxycoumarin; (**9**) cichoric acid; (**10**) apiin; (**11**) *o*-coumaric acid; (**12**) coumarin.

The presence of three flavonoid compounds—verbascoside, apiin and luteolin-7-*O*-glucoside—was observed in the raw material (flowering herb). The content of these compounds strictly depended on both the shading and the developmental stage of the plants. With respect to the light access, the highest content of all the above mentioned flavonoids was detected in the herb collected from the plants grown in full sunlight while the lowest was noted in those grown in deep (50%) shade. The level of verbascoside and apiin was distinctly higher after the seed-setting stage compared to that recorded in the full flowering stage. In the case of luteolin-7-*O*-glucoside, the opposite tendency was observed as the content of this compound was observed to be the highest at the full flowering stage (Table 1, Figure 1).

Coumarin, with its derivatives and phenolic acids, namely 3,4-dihydroxycoumarin, *o*-coumaric acid 2-*O*-glucoside, as well as *o*-coumaric, *p*-coumaric, chlorogenic, caffeic, ferulic, cichoric acids were also found in the plants. Coumarin, a compound that is responsible for the distinct odor of the herb, at the flowering stage was the highest in the plants grown under 30% shade. After the seed-setting stage, its content decreased in the plants from full sunlight and 30% shade. The opposite tendency was observed in the case of plants grown in 50% shade, which showed an increased content of coumarin after the seed-setting stage that was estimated to be the highest among all the variants of investigated plants studied (285.16 mg per 100 g dry weight (DW)). In the plants cultivated in 50% shade, the content of 3, 4-dihydroxycoumarin was similar in both the developmental phases investigated in the study, whereas in those grown in full sunlight and 30% shade the content was higher after the seed formation. The content of *o*-coumaric acid, its 2-*O*-glucoside, and *p*-coumaric acid was higher at the flowering stage; however, their concentrations was dependent on the shading level. Among the phenolic acids, chlorogenic acid was found to be the dominant compound. Its content was threefold higher after the seed-setting stage compared to the flowering stage. Regardless of the development phase, its concentration was the highest in the plants grown in full sunlight and the lowest in those grown in deep (50%) shade. The same relationship was observed in the case of the caffeic, ferulic and cichoric acid (Table 1, Figure 1).

### 2.2. Antioxidant Activity of Plant Extracts

Regardless of the analytical method used, the strongest antioxidant activity was found in the herb collected from the plants grown in full sunlight. When ABTS (2,2′-azino-bis3-ethylbenzothiazoline-6-sulfonic acid) assay was applied the reducing ability of the herb obtained from the plants cultivated in 30% and 50% shade was found to be similar. However, when DPPH (1,1-diphenyl-2-picrylhydrazyl) and FRAP (as ferric reducing antioxidant power) assays were used the reducing ability of the raw materials collected from the plants grown in 30% shade was found to be higher than the one noted in the case of the plants cultivated in 50% shade (Table 2).

### 2.3. Plant Development

At the flowering stage (in May), the highest number of shoots was found in the plants grown in full sunlight (35 shoots per plant), while the lowest number was observed in those grown under 50% shade (8.6 shoots per plant). However, after the seed-setting stage, a part of the shoots withered in the plants grown under full sunlight. A much lower level of withering was noticed in the plants from 30% shade. On the other hand, in the case of plants grown under 50% shade the number of shoots increased significantly after the seed-setting stage. In addition, the shoots were much longer, and the leaf area was almost threefold bigger compared to the plants grown in full sunlight. Moreover, the plants grown under moderate shade produced more glandular trichomes on the leaves compared to those grown in full sunlight and those cultivated in deep shade. Light access also influenced the mass of herb produced by the plants. At the full flowering stage, the highest mass of herb was obtained from the plants grown in full sunlight. However, after this period the shoots of those plants started to wither and after the seed-setting stage the mass of herb became significantly lower. An opposite tendency was observed in the plants grown in shade. For those cultivated in 30% shade no change in the mass of herb was observed, whereas for those grown under 50% shade an increase in the mass from 86.5 g to 120.0 g fresh weight (FW) was observed per plant. The highest content of chlorophyll a and b was found in the plants grown in deep (50%) shade, whereas the lowest was noted in those grown in direct sunlight. Their concentration was distinctly higher at the full flowering stage than that measured after the seed-setting stage (Table 3).

### 2.4. H_2_O_2_ and MDA Content

The highest content of hydrogen peroxide (H_2_O_2_) was observed in the plants grown under full sunlight (522.46 µmol·g^−1^ FW), and the lowest in those subjected to grow in 50% shade (58.92 µmol·g^−1^ FW). Similarly, the concentration of malondialdehyde (MDA) was the highest in the plants grown in full sunlight while it was significantly lower in the shaded plants (30% and 50% shade, Table 3).

### 2.5. Assays of Antioxidant Enzymes

#### 2.5.1. CAT Activity

Catalase (CAT) activity was the highest in the plants grown under full sunlight conditions (1005.65 units (U)); however, it was high also in the plants from 50% shade (813.16 U). The lowest enzyme activity was observed in the plants grown in 30% shade (Table 3).

#### 2.5.2. PPO Activity

Polyphenol oxidase (PPO) activity was also dependent on the growth conditions of the studied plants and was compatible with the content of total polyphenols. The highest activity of this enzyme was observed in the plants grown in full sunlight, while the lowest activity was detected in the plants subjected to 50% shade (Table 3).

## 3. Discussion

### 3.1. Chemical Traits

The raw material collected from *M. melissophyllum* is herb rich in phenolic compounds. Among them *o*-coumaric, protocatechuic, chlorogenic, vanillic, caffeic, syringic, ferulic, sinapic and cinnamic acid, as well as flavonoids, namely apigenin, kaempferol, luteolin, quercetin and myricetin, were earlier reported [10,13,14,25]. According to the study of Skrzypczak-Pietraszek and Pietraszek [14], the dominant flavonoids in *M. melissophyllum* leaves were cynaroside (from 38.00 to 92.00 mg 100 g^−1^ DW) and rutin (from 19.0 to 43.0 mg 100 g^−1^ DW); however, the total content of flavonoids was higher in the leaves than in flowers. In turn, among phenolic acids, *p*-hydroxybenzoic and *p*-coumaric acids were dominating [13]. In our experiment, carried out on plants at the full flowering and after the seed-setting stage, the dominants were verbascoside (from 165.61 to 1301.40 mg 100 g^−1^ DW), luteolin-7-*O*-glucoside, syn. cynaroside (from 133.57 to 1061.23 mg 100 g^−1^ DW) and chlorogenic acid (from 53.37 to 920.95 mg 100 g^−1^ DW) (Table 1). Hence, far, only limited aspects of the accumulation of secondary metabolites, related to seasonal variation, have been focused on in previous studies. According to those results, the highest amounts of flavonoids were found in the herb of *M. melissophyllum* collected in May compared to the samples obtained in September [14] whereas the content of phenolic acids was significantly higher in September [13]. However, these studies were carried out in situ, on wild-growing plants and the external factors influencing the accumulation of phenolics in plants under controlled, cultivation conditions have not yet been investigated. In our study, we investigated the effects of shading on the accumulation of phenolics in the herb of *M. melissophyllum*. The production of secondary metabolites in plants is considered to be influenced by many factors, among which the most important are genetic profile and the ontogenetic development of the plant. In addition, this process may be affected by external factors such as climate, pollution, diseases, pests and edaphic characteristics [18,26]. In general, phenolics serve as a protective substances or attractants that mediate the interactions between plants and the environment. They effectively control certain steps of cell growth and differentiation and thus play an important role in the development and reproduction of plants [18,24,27,28,29]. Therefore, studies aiming at the determination of the factors influencing the accumulation of phenolics may be useful in the production of high-quality herbal raw materials, with the quality being directly related to the presence and content of specific compounds. Our results showed that light access greatly influenced the accumulation of the determined phenolic compounds and the content of total polyphenols. Among these, flavonoids and most phenolic acids were found to be present at the highest level in the herb of plants grown in full sunlight. This was observed especially in the case of verbascoside (from 307.65 mg 100 g^−1^ DW in 50% shade to 1304.40 mg 100 g^−1^ DW in full sunlight; after the seed-setting stage) and luteolin-7-*O*-glucoside (from 133.57 mg 100 g^–1^ DW in 50% shade to 947.65 mg 100 g^−1^ DW in full sunlight; after the seed-setting stage) (Table 1). Such phenomenon is probably associated with the role played by these phenolics in plants. Both flavonoids and phenolic acids are considered to be defense compounds synthesized by plants in response to stress caused by sun exposure. In case of many species it was demonstrated that higher doses of photosynthetically active radiation (PAR), in the range of 400–700 nm, stimulate the synthesis of flavonoids and hydroxycinnamic acids. In particular, this was well documented in shade-loving plants such as bilberry (*Vaccinium myrtillus* L.), *Mikania laevigata* Sch. Bip. ex Baker and *Mikania glomerata* Spreng. [21,30]. Flavonoids can greatly inhibit reactive oxygen species (ROS). The presence of some of these compounds was identified in the chloroplast or nucleus of mesophyll cells, where they serve as scavengers of free radicals and form complexes with Fe and Cu ions that generate ROS. Generally, excess light reduces the activity of antioxidant enzymes in the chloroplast and upregulates the biosynthesis of flavonoids, which are considered to be a “secondary” antioxidant system, preventing the destruction of the photosynthetic system [27,29].

Compared to the data available on flavonoids or phenolic acids in the literature, information about the factors affecting the accumulation of coumarin and its derivatives in plants is scarce. According to Bertolucci et al. [30], the content of coumarin in the leaves of *M. laevigata* increased with the shade level. The same tendency was observed in *Hierochloë australis* (Schrad.) Roem. et Schult. [31]. Similar to *M. melissophyllum*, both species are typical undergrowth plants, found growing on semi shaded sites. Thus, it seems that the accumulation of coumarin in plants is light-specific. In the case of *M. melissophyllum*, the level of coumarin in its herb was also related to its developmental stage. According to Maggi et al. [10] total coumarin content in its leaves may vary during the phenological cycle from 3091 (in July) to 11,125 mg kg g^−1^ DW. In our research, at the flowering stage, its content was the highest when the plants were grown in moderate (30%) shade (268.19 mg 100 g^−1^ DW), whereas after the seed-setting stage it was the highest in plants grown under 50% shade (285.16 mg 100 g^−1^ DW). By contrast, at both the developmental stages, the level of coumarin in plants grown in full sunlight was distinctly lower (Table 1). In the raw materials of *M. melissophyllum* the content of coumarin may be considered as a quality marker since it is known to determine the aroma of the herb. Although this compound, which is suspected to induce liver toxicity, cannot be added as a flavoring agent to foodstuffs, it is present in many plants that are used on a daily basis including tea (*Camellia sinensis* (L.) Kuntze), cinnamon (*Cinnamomum verum* J. Presl), tonka bean (*Coumarouna odorata* Aubl.) and lavender (*Lavandula officinalis* Chaix). In addition, coumarin is valued for its sweet-herbaceous and cherry flower-like odor. Therefore, this compound is used as a fixative and enhancing agent in perfumes and is also added to cosmetics, detergents and tobacco products [32,33].

### 3.2. H_2_O_2_ Content, Antioxidant Enzymes Activity and Antioxidant Activity of Plant Extracts

According to the literature, at the cellular level, environmental stress such as drought and inappropriate radiation or temperature induces the overproduction of ROS, including superoxide (O_2_^−^), singlet oxygen (^•^O_2_), hydroxyl ion (OH^−^), and H_2_O_2_, which are harmful to cell compounds [34]. In order to adopt to detrimental environmental factors, plants utilize their defense systems for scavenging and detoxifying ROS through enzymes such as CAT, peroxidase or superoxide dismutase and decompose H_2_O_2_ to H_2_O at different cellular locations [35,36,37]. In our study, the highest H_2_O_2_ content—one of the most important ROS was observed in the plants grown under full sunlight conditions which also exhibited the highest CAT and PPO activities. An interesting fact is, that the content of H_2_O_2_ in the tested plants was not associated with their CAT activity, which in turn was found to be high in the plants subjected to 50% shade (813.16 U). This phenomenon may be a result of the high CAT activity in the earlier developmental phases of the studied plants and the same probably with high H_2_O_2_ content (Table 3). A low H_2_O_2_ was found in 50% shaded plants, which may be a result of the perturbations in the CAT activity and the overproduction of this enzyme. However, further analyses should be performed to confirm this hypothesis.

Thus far, a number of methods have been developed for determining the antioxidant activity. Usually, these methods analyze the activity of a specific group of compounds. Therefore, for determining the antioxidant activity of plant extracts different methods are applied in parallel. Among these, DPPH and ABTS radical scavenging methods are the most popular and most commonly used due to their sensitivity and ease [38]. These methods allow determining the activity of both hydrophobic and hydrophilic compounds [39,40]. In turn, antioxidants that react in the FRAP analysis can reduce the Fe^3+^ TPTZ salt into Fe^2+^ TPTZ form. These includes vitamin C and E and some phenolic compounds as well [40]. According to our results, regardless of the method used, the highest antioxidant activity was found in herb obtained from the *M. melissophyllum* plants grown in full sunlight (Table 2). This finding was much related to the content of phenolic compounds in this herb, rich in flavonoids and phenolic acids (Table 1). The antioxidant activity of *M. melissophyllum* extracts depends also on the extraction method applied [41]. The activity of methanolic and ethanolic extracts was found to be similar but was lower than that of ethyl acetate or chloroform extracts. However, the authors reported that the concentration of ethanol influenced the antioxidant activity. Numerous publications on Lamiaceae species indicate on their strong free radical scavenging activity which is associated with their high phenolics content. Among these species, *M. melissophyllum*, grown in specific environmental conditions, seems to be an interesting source of antioxidants.

### 3.3. Plant Development

Thus far, little is known about the developmental biology of *M. melissophyllum*, including its reproductive mechanisms and the factors that affect its growth. Some data on its morphology can be found in general botanical elaborations concerning the Lamiaceae family [1]. The species originates in Europe and is a typical understory plant found growing wild in mixed forests [1,2]. Shade seems to be one of the most important factors influencing its development. Generally, the response of plants to shade is considered to strictly depend on their genotype, and thus is species-specific [19]. An inappropriate light intensity damages the photosynthetic system of the plant, which in turn affects their photosynthesis and development. Our study showed that when grown in direct sunlight, *M. melissophyllum,* a typical shade-loving plant, produced more number of shoots than those grown in shade; however, the shoots were relatively short and the leaf area of those plants was 2–3 times smaller than those grown in shade (Table 1). Leaves are the main light-acquiring organs so when a plant subjected to acclimatize to inhospitable conditions, the structure and diameters of the organ may change [42,43,44]. Usually, when grown under deep shade, the leaf area increases in order to increase light acquisition, while its thickness and mass per unit are reduced [45]. In the present study, it was observed that when *M. melisophyllum* plants were grown in 30% shade, their leaves were covered with a higher number of glandular trichomes, responsible for the accumulation of essential oils (Table 3). These compounds are considered to be the most important attractants of pollinators. The presence and structure of glandular trichomes on the vegetative and reproductive organs of this plant were previously reported by Maggi et al. [9]. The specific type of plant odor allows the insects to discriminate between the flower species and begin the behavioral reaction that results in pollination [26]. Thus, the essential oils produced in glandular trichomes of *M. melissophyllum* leaves may contribute to the reproductive success of the plant.

## 4. Materials and Methods

### 4.1. Field Experiment

Field experiment was carried out at the Experimental Station of Warsaw University of Life Science (WULS-SGGW), in the years 2016–2019. The seeds of *M. melissophyllum* used to establish the experiment were obtained in situ, from the population growing in Eastern Poland (near Koryciny village, N52°37.944’ E22°45.718’). The seedlings were produced in the greenhouse of WULS–SGGW and were planted out at a spacing of 50 × 40 cm at the beginning of October 2016. The plants were cultivated on a medium-heavy alluvial soil amended with peat of pH _(KCl)_ 6.5 and river sand. A part of these were grown in full sunlight (0%), while the other were cultivated under polypropylene fabrics that reduced the light intensity by 30% and 50%. The experiment had three replications, with 35 plants grown per plot and was conducted on 3-year-old plants.

### 4.2. Harvest of Raw Material

The raw material (herb) was collected at two developmental stages—at full flowering (May) and after seed-setting (July). The intensity of photosynthetic active irradiation (PAR; µmol photons m^−2^ s^−1^) was measured in both these stages (Table 4). The herb was cut at 5 cm above the ground level from ten randomly selected plants in three replications and dried at 35 °C in the dark. FW and DW of the herb were also determined.

### 4.3. HPLC-DAD Analysis

The standards applied in high-performance liquid chromatography (HPLC) were purchased from ChromaDex (California, LA, USA), while methanol and acetonitrile (HPLC grade) were obtained from Merck KGaA (Darmstadt, Germany). The extraction of air-dried herb, separation of the analyzed chemical compounds, and validation of the method were carried out as described earlier by Bączek et al. [46]. The analysis were carried out as follows: 1.000 g of air-died finely powdered raw material was extracted with 100 mL of methanol in Extraction System B-811 (Büchi Labortechnik AG, Flawil, Switzerland). After evaporation of the solvent, the residue was dissolved in 10 mL of methanol, filtered with Supelco Iso-Disc^™^ Syringe Tip Filter Unit, PTFE membrane (Merck KGaA, Darmstadt, Germany) and subjected to HPLC analysis.

Quantitation was performed at a wavelength appropriate for each substance: 276 nm for coumarin, 3,4-dihydroxycoumarin, *o*-coumaric acid and *o*-coumaric acid 2-*O*-glucoside; 309 nm for *p*-coumaric acid; 325 nm for chlorogenic, caffeic, ferulic and cichoric acid; 330 nm for verbascoside; 336 nm for apiin; and 347 nm for luteolin-7-*O*-glucoside. The standard curve parameters and validation data were calculated with Microsoft Excel (Table 5).

### 4.4. H_2_O_2_ Content and Lipid Peroxidation

The H_2_O_2_ content of leaves was measured spectrophotometrically following reaction with potassium iodide (KI) as described by Jędrzejuk et al. [47]. Absorbance was measured at 390 nm and the results were expressed as µmol of H_2_O_2_ g^−1^ FW.

Lipid peroxidation was measured as the amount of MDA produced by the reaction of thiobarbituric acid (TBA) according to the method of Hodges et al. [48] with some modifications described for common lilac petals by Jędrzejuk et al. [47]. The MDA content was expressed as µmol MDA g^−1^ FW.

### 4.5. Assays of Antioxidant Enzymes

#### 4.5.1. CAT Activity

CAT (EC 1.11.1.6) activity was determined spectrophotometrically as the rate of disappearance of H_2_O_2_ at 405 nm, according to the method described by Goth [49] and modified by Jędrzejuk et al. [48] for petals of common lilac. The enzyme activity was expressed as U g^−1^ FW. One unit of CAT deoxidizes 1 µmol of H_2_O_2_ in 1 min.

#### 4.5.2. PPO Activity

PPO activity was measured according to the method of Jariteh et al. [50]. Briefly, 0.1 mL of crude enzyme, 3.9 mL phosphate buffer (pH 6.0) and 1 mL of 0.l M aqueous catechol were mixed in a 30 °C water bath for 10 min. Then, 2 mL of 20% trichloroacetic acid was added quickly to stop the reaction. The absorbance of PPO was recorded immediately at 525 nm. One unit of PPO activity is equivalent to an increase in 0.01 times the amount of enzyme for 1 g FW in 1 min.

### 4.6. Antioxidant Activity of Plant Extracts

For analyzing the antioxidant activity, the raw materials collected at the full flowering stage were used. DPPH and ABTS scavenging capacity assays as well as FRAP assay were applied. 0.25 g of finely powdered air-dried raw material was extracted in 5 mL of methanol per 60 min using ultrasound extraction. The extracts were filtered with Supelco Iso-Disc^™^ Syringe Tip Filter Unit, PTFE membrane (Merck KGaA, Darmstadt, Germany) and subjected to the analysis previously described in detail by Kosakowska et al. [51].

### 4.7. Developmental Traits

During two developmental stages—at full flowering (May) and after seed-setting (July)—the following traits were measured: The number of shoots per plant, their length and the number of nodes per shoot. In addition, at the flowering stage, the leaf area, and the number of glandular trichomes on the leaf were assessed. These measurements were carried out on one shoot per five randomly selected plants, in three repetitions. The leaves of each individual plant were scanned at a resolution of 300 dpi using a flatbed scanner and the leaf area was measured using Leaf Area Meter AM100 (ADC Bioscientific Ltd., Hoddesdon, UK).

The number of glandular trichomes was measured in an area of 1 cm^2^ under the surface of a leaf, in three replications per leaf. This assessment was carried out on the leaves located at three different levels on the shoot—upper, mid-stalk and bottom. The measurements were taken using SMZ745T stereoscopic microscope (Nikon, Tokyo, Japan), with Invenio 3S (3M Pixel CMOS) camera (DeltaPix, Smorum, Denmark) and Coolview v. 1.4 software (Precoptic Co, Warsaw, Poland).

The content of both chlorophyll a and b in the air-dried herb was assessed according to the method described by Lichtenthaler and Wellburn [52].

## 5. Conclusions

*Melittis melissophyllum* is an endangered species used as a medicinal and aromatic plant. Our results showed that shade level significantly affects the accumulation of biologically active compounds in this plant. The plants grown in full sunlight produced 2–3 times more flavonoids and phenolic acids, compared to those grown in shade. However, in 30% and 50% shade they accumulated more coumarin, the compound considered as a quality marker of *M. melissophyllum* herb, determining its specific, pleasant aroma. The optimal condition that promotes its development seems to be moderate (30%) shade. In turn, the plants in full sunlight experienced stress, which was confirmed by the content of H_2_O_2_, activity of antioxidant enzymes, level of chlorophyll a and b and the content of photosynthesis-related secondary metabolites in its herb. Thus, the results of the present study indicate that cultivation under appropriate shading not only promotes the development of this plant, but also modulates the quality of the obtained raw materials. These results may be useful for adopting suitable actions to protect the natural resources of *M. melissophyllum*, as well as for the introduction of this valuable, medicinal plant into cultivation and forecasting the quality of its herb.

## Figures and Tables

**Table 1:** Effects of plant shading on the content of phenolic compounds in the herb (mg 100 g^−1^ dry weight (DW)).

Compounds	Developmental Stage	Shade Level					
		0%		30%		50%	
Flavonoids							
Verbascoside	full flowering	439.51	±31.51 a	283.43	±34.60 b	165.61	±23.00 c
	after seed-setting	1301.40	±23.00 a *	614.66	±27.64 b *	307.65	±30.20 c *
Apiin	full flowering	91.17	±5.49 a	26.09	±4.96 b	6.17	±1.11 c
	after seed-setting	197.90	±9.65 a *	88.26	±5.49 b *	38.29	±6.94 c *
Luteolin-7-*O*-glucoside	full flowering	1061.23	±63.56 a *	432.95	±33.60 b	183.05	±29.10 c
	after seed-setting	947.65	±29.02 a	535.63	±30.00 b *	133.57	±23.90 c
Coumarins and phenolic acids							
Coumarin	full flowering	221.36	±11.60 b *	268.19	±12.36 a *	224.65	±13.60 b
	after seed-setting	109.83	±13.50 c	163.86	±13.10 b	285.16	±17.30 a *
3.4-Dihydroxycoumarin	full flowering	88.28	±5.30 a	49.23	±2.30 b	55.78	± 3.60 b *
	after seed-setting	149.96	±7.80 a *	67.22	±6.32 b *	47.27	±1.68 c
*o*-Coumaric acid	full flowering	60.83	±4.41 b *	144.12	± 10.02 a *	31.27	±6.20 c
	after seed-setting	16.6	±2.13 b	14.54	±0.23 b	26.14	±3.46 a
*o*-Coumaric acid 2-*O*-glucoside	full flowering	423.42	±44.36 a *	316.61	±35.00 b *	238.34	±25.10 b *
	after seed-setting	115.77	±21.30 a	102.35	±24.80 a	111.91	±19.30 a
*p*-Coumaric acid	full flowering	3.38	±0.28 a *	3.46	±0.31 a *	2.83	±0.67 a *
	after seed-setting	1.40	±0.10 a	0.87	±0.13 b	0.91	±0.16 b
Chlorogenic acid	full flowering	275.79	±15.60 a	152.95	±18.60 b	53.37	±12.60 c
	after seed-setting	920.95	±35.20 a *	528.18	±26.30 b *	170.68	±28.00 c *
Caffeic acid	full flowering	9.76	±0.95 a	3.20	±0.98 b	2.54	±0.36 b *
	after seed-setting	14.06	±1.12 a *	4.40	±1.01 b	1.28	±0.12 c
Ferulic acid	full flowering	9.33	±0.69 a	5.85	±1.30 b	5.21	±1.20 b
	after seed-setting	77.66	±16.31 a *	20.17	±13.60 b	11.42	±4.50 b
Cichoric acid	full flowering	107.80	±16.12 a	50.40	±1.12 b	22.87	±1.93 b
	after seed-setting	224.14	±31.82 a *	69.38	±12.30 b *	21.76	±2.60 b

Values are mean ± SD; values marked in rows with different letters differ at *p* < 0.05;* *p* < 0.05 (in columns).

**Table 2:** Antioxidant activity of extracts obtained from the plants grown under various shades.

Method		Shade Level					
		0%		30%		50%	
DPPH	(%RSC)	90.41	±1.00a	53.17	±1.00b	46.86	±0.98c
	(µmol Trolox/g)	312.17	±1.10a	235.15	±1.00b	157.61	±1.01c
ABTS	(%RSC)	87.61	±1.00a	49.95	±0.95b	52.45	±1.30b
	(µmol Trolox/g)	401.75	±1.20a	256.00	±1.12b	256.40	±1.00b
FRAP	(Fe^2+^µmol/g )	86	±1.00a	34	±1.00b	26	±1.00c
	(µmol Trolox/g)	967	±2.09a	384	±1.11b	252	±1.07c

Values are the mean ± SD; values marked in rows with different letters differ at *p* < 0.05.

**Table 3:** Effects of plant shading on selected developmental and physiological traits.

Traits	Developmental Stage	Shade Level					
		0%		30%		50%	
number of shoots per plant	full flowering	35.2	±8.4 a *	20.2	±9.0 b	8.6	±2.8 b
	after seed-setting	20.2	±8.3 a	17.4	±5.9 ab	12.8	±4.8 b
length of shoots (cm)	full flowering	32.2	±2.6 b	36.0	±1.7 ab	36.9	±3.4 a
	after seed-setting	32.0	±5.9 b	39.1	±4.9 ab	45.3	±8.0 a
number of nodes per shoot	full flowering	6.2	±0.4 b *	7.8	±0.5 a *	7.0	±1.0 ab
	after seed-setting	5.2	±0.6 b	6.5	±0.6 a	7.2	±1.0 a
leaf area (mm^2^)	full flowering	2573.20	±313.25 c	4587.20	±663.73 b	7182.00	±510.68 a
number of glandular	upper leaf	38.33	±4.51 a	39.78	±1.97 a	41.78	±6.38 a
trichomes per cm^2^	mid-stalk leaf	36.11	±4.03 b	46.30	±4.71 a	24.11	±3.39 c
	bottom leaf	19.44	±3.00 b	59.89	±6.87 a	22.23	±2.61 b
fresh weight (FW) of herb (g plant ^−1^)	full flowering	162.9	±37.3 a *	129.4	±39.4 ab	86.5	±30.2 b
	after seed-setting	100.1	±14.4 a	130.1	±41.8 a	120.0	±27.4 a *
dry weight (DW) of herb (g plant ^−1^)	full flowering	28.9	±10.7 a	17.9	±4.9 ab	14.0	±3.6 b
	after seed-setting	20.9	±5.0 ab	23.7	±5.8 a	15.0	±3.5 b
chlorophyll a	full flowering	3.48	±0.36 b *	4.51	±0.32 a *	4.54	±0.25 a *
(μg g^−1^ DW)	after seed-setting	2.49	±0.28 b	3.87	±0.25 a	4.00	±0.29 a
chlorophyll b	full flowering	1.52	±0.13 b *	1.90	±0.11 a *	2.00	±0.15 a *
(μg g^−1^ DW)	after seed-setting	0.84	±0.08 b	1.62	±0.07 a	1.82	±0.06 a
H_2_O_2_ (µmol g^−1^ FW)	after seed-setting	522.46	±60.05 a	150.92	±8.07 b	58.92	±7.90 c
MDA (µmol g^−1^ FW)	after seed-setting	24.01	±1.42 a	20.09	±1.32 b	21.43	±1.62 b
CAT (U g^−1^ FW)	after seed-setting	1005.62	±289.5 a	205.04	±25.50 c	813.16	±78.90 b
PPO (U g^−1^ FW)	after seed-setting	154.62	±17.66 a	131.11	±12.79 b	67.19	±10.88 c

Values are mean ± SD; values marked in rows with different letters differ at *p* < 0.05; * *p* < 0.05 (in columns).

**Table 4:** The intensity of photosynthetically active radiation (PAR) (µmol photons m^−2^ s^−1^).

Developmental Stage	Shade Level		
	0%	30%	50%
flowering stage (May)	2420	1650	720
after seed-setting (July)	2600	1770	850

**Table 5:** HPLC-DAD validation parameters (*n* = 6).

Compound	Wavelength (nm)	Precision Intra-day (CV)	Precision Inter-day (CV)	Regression Equation	R2 (*n* = 6)	Linear Range (µg × mL^−1^)	LOD (µg × mL^−1^)	LOQ (µg × mL^−1^)	Recovery (%)
3-*O*-Caffeoylquinic acid (Chlorogenic acid)	325	1.32	1.63	y = 6517.4 x − 12,017	0.999	0.40–39.46	0.21	0.70	98.5
3,4-Dihydroxycinnamic acid (Caffeic acid)	325	1.00	1.72	y = 2592.9 x + 380	0.999	1.00–998.40	0.03	0.08	96.8
*o*-Coumaric acid 2-*O*-glucoside (*trans*-Melilotoside)	276	0.66	1.32	y = 5143.3 x − 472	0.999	0.11–36.42	0.01	0.03	104.2
4-Hydroxycinnamic acid (*p*-Coumaric acid)	309	0.28	0.65	y = 6196.4 x − 538	0.999	1.01–504.70	0.70	0.23	101.9
Verbascoside	330	0.68	0.98	y = 2638.9 x − 596	0.999	0.21–205.63	0.05	0.17	98.2
Luteolin-7-*O*-glucoside	347	2.36	2.67	y = 2022.2 x − 1149	0.999	0.19–19.08	0.05	0.18	101.4
4-Hydroxy-3-methoxycinnamic acid (Ferulic acid)	325	0.58	0.84	y = 2424.6 x − 1857	0.999	0.40–399.68	0.11	0.35	102.3
3,4-Dihydroxycumarin	276	0.57	0.93	y = 1275.6 x − 529	0.999	0.38–126.67	0.03	0.11	97.5
Cichoric acid	325	0.18	0.49	y = 3230.7 x + 6882	0.999	0.46–456.96	0.11	0.38	102.8
Apigenin-7-O-apioglucoside (Apiin)	336	0.88	1.27	y = 2757.8 x − 1063	0.999	0.69–690.12	0.12	0.40	101.2
*o*-Coumaric acid	276	0.65	1.12	y = 5304.8 x − 4725	0.999	1.06–353.08	0.01	0.03	102.5
Coumarin	276	0.64	0.98	y = 3975.2 x − 1811	0.999	0.40–133.20	0.01	0.04	98.8
